# Physical Activity and Body Composition Are Associated With Severity and Risk of Depression, and Serum Lipids

**DOI:** 10.3389/fpsyt.2020.00494

**Published:** 2020-06-05

**Authors:** Claudia von Zimmermann, Merle Winkelmann, Tanja Richter-Schmidinger, Christiane Mühle, Johannes Kornhuber, Bernd Lenz

**Affiliations:** ^1^Department of Psychiatry and Psychotherapy, Friedrich-Alexander University Erlangen-Nürnberg (FAU), Erlangen, Germany; ^2^Department of Addictive Behavior and Addiction Medicine, Central Institute of Mental Health (CIMH), Medical Faculty Mannheim, Heidelberg University, Mannheim, Germany

**Keywords:** depression, physical activities and sports, body composition, serum lipid concentration, mental health

## Abstract

**Background:**

Physical activity and a healthy body composition are said to reduce the risk of major depressive disorder. Nonetheless, deeper insight is needed into which specific forms of physical activity (and their relation to body composition) are effective in improving and preventing depressive symptoms.

**Methods:**

We compared different self-reported physical activities of the Global Physical Activity Questionnaire and body composition measures between patients with a current major depressive episode (MDE; *N* = 130) and healthy control subjects (*N* = 61). These parameters were also tested for correlations with depression severity and serum lipid levels in patients and controls.

**Results:**

Patients with a current MDE reported significantly fewer hours spent on total physical activity, walking or bicycling for travel, and vigorous-intensity activities at leisure than healthy control subjects. More time spent on vigorous-intensity activities at work, less time spent on walking or bicycling for travel, higher body fat mass, and lower body muscle mass correlated significantly with stronger depression severity. Physical activity and body measures correlated significantly with serum lipid levels.

**Limitations:**

Self-reports of physical activity, only short-term follow-up of 20 days, cross-sectional study design without examination of causal role of exercise.

**Conclusions:**

More time spent on traveling by foot or by bike is especially associated with a lower risk of and milder depression. These results highlight the differential role of physical activity in depression.

## Introduction

Major depressive disorder (MDD) is one of the five leading causes for years lived with disability (1), and 30% of the depressed patients are considered therapy refractory. Suffering from depression entails immense individual burden and creates high economic costs for society. Hence, there is urgent need for effective prevention strategies.

The World Health Organization (WHO) recommends at least 150 min of moderate-intensity physical activity throughout the week to reduce the risk of noncommunicable diseases (NCDs) like depression (2). Some studies show a reduction of depressive symptoms following resistance exercise training (3). However, it seems that sport administered in a therapeutical setting has only a minor positive impact on depressive symptoms overall (4). Instead, physical activity appears to be more effective in prevention of depression. In contrast to physical activity at work, physical activity during leisure-time might protect against the development of a depressive disorder depending on the amount but regardless of the intensity (5). Using the genetic instrument of Mendelian randomization, Choi et al. (6) clarified that reduced physical activity is a risk factor for depressive symptoms, not only a consequence. Overall, we have only limited knowledge about which subforms of physical activity are likely to help preventing depression.

Physical activity is also important for weight control and prevention of obesity. On the one hand, especially the atypical subtype of MDD is a strong predictor of obesity and weight gain in the future (7). On the other hand, obesity increases the risk of future depression (8). Higher body mass index (BMI) was associated with increased risk of depression in a large prospective population survey (9). In addition, abdominal fat distribution may be a key mediator in the relationship between obesity and depression (10). Based on these data, we would expect that patients with a current major depressive episode (MDE) show body measures related to obesity, i.e., primarily a higher BMI.

Moreover, physical activity reduces the likelihood of NCDs and depressive comorbidities, particularly cardiovascular disease (11). There is evidence that physical training improves cholesterol metabolism, e.g., lowers LDL lipoprotein levels (12, 13). Cholesterol metabolisms and serum lipids seem to play an important role in depression. It has been shown that high triglycerides, high total cholesterol, and high LDL cholesterol are related to depression *per se* (14). By contrast Ancelin et al. (15) found out that lower LDL cholesterol is a risk factor for depressive symptoms in elderly males. Using lipid lowering statins as an additional medication to antidepressants, Salagre et al. (16) found a better Hamilton Depression Rating Scale (HAMD) outcome in patients compared to those with antidepressants and placebo in a large meta-analysis of epidemiological studies. Interestingly, cerebral lipids, especially ceramides, have been shown to be a potential target of antidepressants and influence depression like behavior in animal experiments (17). Considering the possible LDL lowering effect of physical activity, lipid pathways might thus be involved in the relationship between physical activities and depression.

In this study, we aimed to investigate which physical activities in everyday life (total physical activity, vigorous- and moderate-intensity activities at work and at leisure, and time spent walking or bicycling for travel; sitting as a control condition) are associated with the risk and severity of depressive disorders. We further analyzed which related body composition measures (BMI, body weight and height, waist circumference, body fat and muscle mass, and visceral adipose tissue) are associated with the risk and severity of depressive disorders. We also analyzed the association of physical activity with serum lipids. We have previously found in the same cohort that higher serum lipids, especially higher LDL cholesterol, are positively associated with depression severity (18).

Sex differences in depression, like the two-fold increased lifetime prevalence in women compared with men, are well known (19). Obesity seems to be more prevalent in women. Worldwide, 11% of all men and 15% of all women are classified as obese (20). Li et al. (21) found a positive correlation between depression and BMI in women, but not in men.

Hagströmer et al. (22) showed men spending more time than women in moderate and vigorous physical activity, but no sex-specific differences for inactivity in a large population based study.

Based on these sex differences, we also explored whether the effects were present in analyses specific to women and men.

Our primary hypothesis was that more time spent on physical activity is associated with a lower risk of a current MDE and lower depression severity. Second, we hypothesized that lower serum lipids, especially LDL cholesterol, are associated with protective physical activities. Third, we hypothesized that higher body composition measures, especially higher BMI and visceral adipose tissue, are positively associated with higher risk of depression.

## Methods

### Sample Description

We used data from participants of the CeraBiDe (“Ceramide-associated Biomarkers in Depression”) study (18, 23). The recruitment took place between 01/2014 and 01/2017. Patients with a current MDE were recruited from in- and outpatients of the Department of Psychiatry and Psychotherapy at the University Hospital Erlangen and further interested persons fulfilling the inclusion criteria. Healthy control subjects were local citizens informed about the study *via* letters, local newspapers, flyers, and *via* internet advertisement; we matched both groups by age and gender. Participants underwent a multi-step screening procedure to exclude severe physical (e.g., autoimmune disorder, cancer) and psychiatric morbidity (with the exclusion of nicotine dependence and comorbid anxiety disorder), and the use of corticosteroids or anti-inflammatory drugs in the last 7 days, pregnancy, and breastfeeding (see **Supplementary Material**). The study sample characteristics are provided in **Table 1**. In total, we included 130 patients with a current MDE (64 without any antidepressants for at least 2 weeks and 66 persons taking antidepressants in a stable regime for at least 2 weeks) and 61 healthy control subjects. Inclusion criteria were BMI 18.5–35 kg/m², age 18–75 years, and written informed consent. We screened all participants using a shortened German version of the structured clinical interview for DSM-IV (SCID-I) and quantified depression severity using the self-report Beck Depression Inventory (BDI)-II and the clinician-administered 17-item HAMD. The patients had to fulfill the criteria for a MDD in the SCID-I.

**Table 1:** Study cohort and group comparisons.

	Patients with a current MDE				Healthy control subjects					Group comparisons	
	*N*	% or Median	IQR		*N*	% or Median	IQR			χ^2^, df ^a^ or *U* ^b^	*P*
Female (%)	130	53			61	51			^a^	0.1, 1	0.771
Single (%)	130	38			61	23			^a^	4.1, 1	**0.043**
Married (%)	130	40			61	33			^a^	0.9, 1	0.338
Divorced (%)	130	21			60	5			^a^	7.7, 1	**0.006**
Age (years)	130	46	33	54	61	42	32	54	^b^	3738	0.523
Sum of education years	115	15	13	17	51	15	13	18	^b^	2648	0.317
Paid working hours per week	119	24	0	40	51	25	0	40	^b^	3015	0.946
Paid working months during the previous year	120	12	3	12	53	12	1	12	^b^	3036	0.594
Depression scores											
BDI-II score at baseline	130	29	23	34	61	1	0	3	^b^	10	**<0.001**
BDI-II score at follow-up	120	20	14	28							
HAMD score at baseline	130	22	19	25	61	1	0	2	^b^	0	**<0.001**
HAMD score at follow-up	120	18	12	21							
Physical activity											
Total physical activity (min/week)	127	420	180	1260	61	870	540	1680	^b^	2567	**<0.001**
Vigorous-intensity activities at work (min/week)	130	0	0	0	61	0	0	0	^b^	3706	0.229
Moderate-intensity activities at work (min/week)	129	0	0	360	61	0	0	240	^b^	3878	0.853
Time spent walking or bicycling for travel (min/week)	130	60	0	180	61	210	120	315	^b^	2193	**<0.001**
Vigorous-intensity activities at leisure (min/week)	130	0	0	30	61	120	0	240	^b^	2180	**<0.001**
Moderate-intensity activities at leisure (min/week)	128	60	0	180	61	90	0	210	^b^	3342	0.096
Sitting (min/day)	130	480	300	660	61	420	300	600	^b^	3517	0.206
Body measures											
BMI (kg/m^2^)	130	27	23	29	61	24	23	28	^b^	3335	0.077
Body height (cm)	130	172	166	180	61	172	167	183	^b^	3613	0.322
Body weight (kg)	130	80	67	90	61	73	67	89	^b^	3717	0.486
Waist circumference (cm)	123	91	80	101	57	88	82	95	^b^	3011	0.128
Body fat mass (%)	127	32	26	39	59	29	22	36	^b^	3122	0.068
Body muscle mass (%)	127	30	26	33	59	32	27	36	^b^	3100	0.058
Visceral adipose tissue (%)	127	9	6	11	59	7	5	10	^b^	3117	0.064

MDE, major depressive episode; IQR, interquartile range; BMI, body mass index. N, total number of individuals with data for these parameters. ^a^χ^2^ test; ^b^Mann-Whitney U test. P < 0.05 in bold print.

One hundred twenty of the 130 patients with a current MDE participated in a direct follow-up (study visit 2) [14–63 days post inclusion, median 20 days, interquartile range (IQR) 16–27]. All patients received treatment as usual during the follow-up period. At the first time point (study visit 1), blood, clinical, behavioral, and body composition parameters [including SCID-I, BDI-II, HAMD, Global Physical Activity Questionnaire (GPAQ), body composition] were collected. During the follow-up visit (study visit 2), a blood taking and a second clinical interview (including BDI-II and HAMD) took place.

### Physical Activity

Physical activity was measured by the GPAQ (24, 25). Participants were asked about the time spent on different types of physical activity in a typical week in the last six months. Total physical activity per week was calculated using metabolic equivalents to activity levels according to the recommendations of the WHO (26). Moderate physical activity at leisure includes activities with moderate physical effort, which cause small increases in breathing or heart rate such as brisk walking, cycling, swimming, or volleyball; vigorous-intensity activities at leisure require hard physical effort and cause large increases in breathing or heart rate, like running or football. Vigorous-intensity activities at work include carrying or lifting heavy loads, digging, and construction work. Moderate-intensity activity at work causes small increases in breathing or heart rate such as brisk walking or carrying light loads.

For the judgment of the GPAQ, it is important to equalize vigorous-intensity to moderate-intensity physical activity. As recommended by the WHO, we doubled the time spent with vigorous-intensity activity to equalize it with moderate level activity. Time spent traveling is considered a moderate activity. Total physical activity means the sum of all single activities per week (without sitting).

### Body Composition

For body composition analysis (BMI, body weight and height, waist circumference, body fat and muscle mass, visceral adipose tissue), a classical measuring tape and body analyzer (Omron BF511, Krell Precision, Jiangsu, China) applying bio-impedance measurement were used.

### Blood Analysis

Blood samples were taken (after fasting overnight) in the morning. Serum lipid levels were quantified at the Central Laboratory of the University Hospital Erlangen, Germany (DIN EN ISO 15189 accredited) by enzymatic photometric assays.

### Statistical Analyses

The data were analyzed using SPSS for Windows 24.0 (SPSS Inc., Chicago, IL) and visualized using Graph Pad Prism 5 (Graph Pad Software Inc., San Diego, CA). The descriptive statistics report median, IQR, and frequencies. The χ^2^ test was used to evaluate differences in the frequency of nominal variables. Because the Kolmogorov-Smirnov test showed significant deviations from normal distributions in most variables of interest in patients with a current MDE and/or healthy control subjects (all depression scores, all times spent on physical activity, all body measures except for body fat mass), we decided to use non-parametric methods. Group differences were tested using the Mann-Whitney *U* tests for independent samples. Spearman's method was applied to evaluate bivariate correlations. *P* < 0.05 for two-tailed tests was considered significant. All P-values are uncorrected for multiple hypothesis testing. We used Receiver Operating Characteristic (ROC) and Youden's J statistic (J = sensitivity + specificity − 1) to evaluate the thresholds of hours of physical activity per week to best discriminate patients with a current MDE from healthy control subjects (27). The results of sex-specific analyses are shown in the **Supplemental Tables**.

### Ethical Approvement

This study was approved by the Ethics Committee of the Medical Faculty of the Friedrich-Alexander University Erlangen-Nürnberg (ID 148_13 B).

## Results

### Sociodemographic Characteristics

We did not find any significant between-group differences in age, sex, education, and time spent working (i.e., paid hours at work per week and paid months during the previous year). Patients with a current MDE were significantly more frequently divorced (OR 5.0) and significantly more often single (OR 2.0) than controls. They also scored significantly higher on the BDI-II and HAMD scores (**Table 1**).

### Between-Group Differences in Physical Activity and Body Measures

Patients with current MDE spent significantly less time on total physical activity, on traveling to and from places by bike or by foot, and on activities with vigorous physical effort in leisure time than healthy control subjects (**Table 1**). Neither time invested in activities at work (vigorous-intensity and moderate-intensity), nor moderate intensity activities at leisure, varied significantly between healthy control subjects and patients with a current MDE. There was also no significant difference in our control parameter, the daily time used for sitting. Moreover, the groups did not significantly vary in body composition measurements (**Table 1**). Most of the effects seen in the total cohort were also present in the sex-specific sub-groups. In addition, we found longer sitting in female and longer vigorous-intensity activities at work in male patients with a current MDE than in sex-specific healthy control subjects. Moreover, male patients with a current MDE showed significantly higher body fat mass and visceral adipose tissue and lower body muscle mass (**Supplemental Tables 1** and **2**).

### Activity Thresholds Discriminating Between Patients with an MDE and Healthy Control Subjects

**Figure 1:**
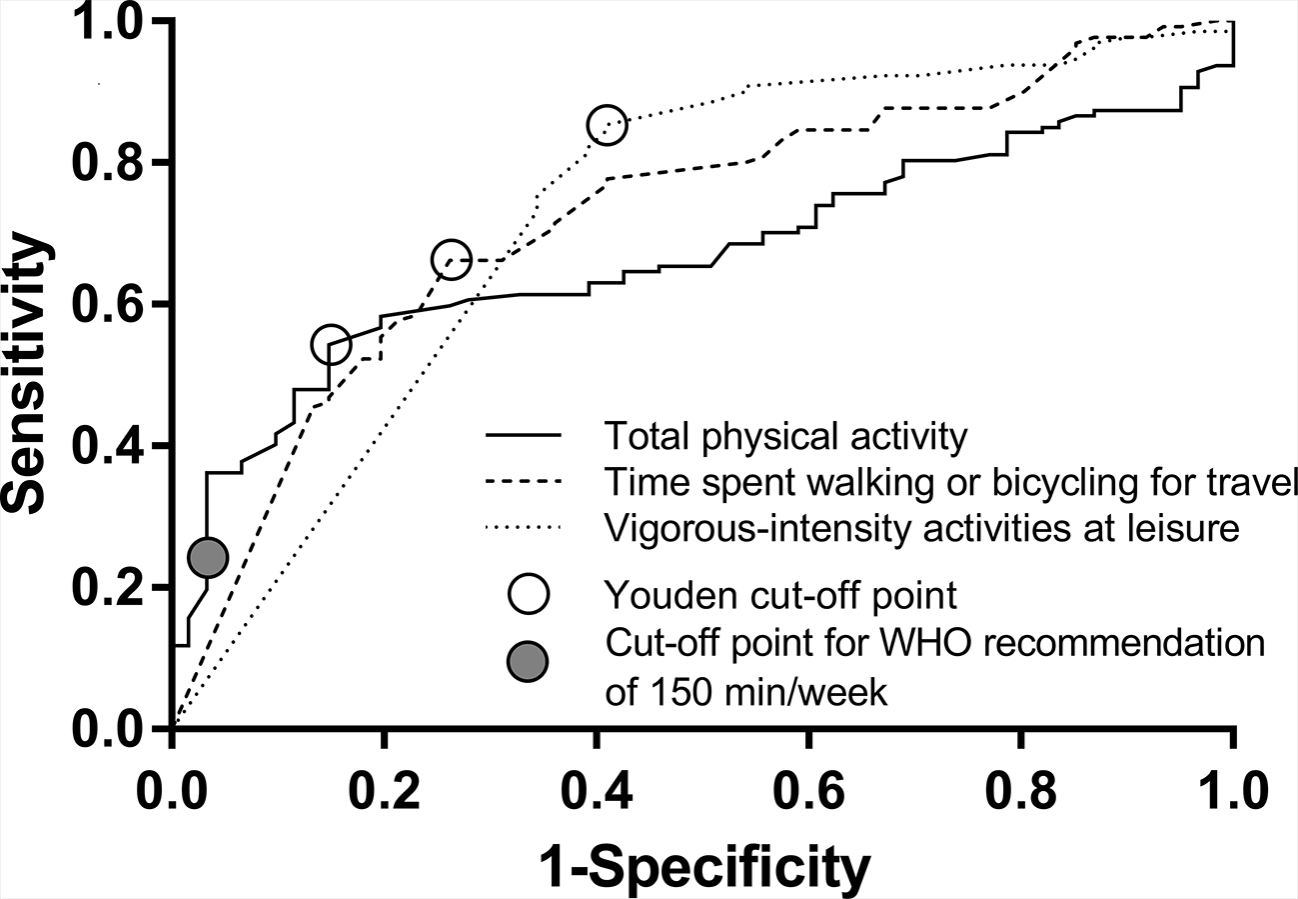
The results of the Receiver Operating Characteristic analysis with open circles indicating Youden's cut-off points.

Because of significant differences in the between-group comparisons for total physical activity, time spent walking or bicycling for travel, and vigorous-intensity activities at leisure, we further conducted ROC analyses to evaluate the thresholds of minutes per week to discriminate patients with MDE from healthy control subjects. These showed Youden cut-off points of 465 min per week of total physical activity (*N* = 188, AUC 0.669, *P* < 0.001, sensitivity 0.543, specificity 0.852), of 123 min per week spent walking or bicycling for travel (*N* = 191, AUC 0.724, *P* < 0.001, sensitivity 0.662, specificity 0.738), and of 113 min per week with vigorous-intensity activities at leisure (*N* = 191, AUC 0.725, *P* < 0.001, sensitivity 0.854, specificity 0.590) (**Figure 1**). In sex-specific analyses, we found Youden cut-off points for total physical activity of 245 min per week in females (*N* = 97, AUC 0.683, *P* = 0.004, sensitivity 0.379, specificity 0.968) and 450 min per week in males (*N* = 91, AUC 0.646, *P* = 0.024, sensitivity 0.492, specificity 0.967). We found Youden cut-off points for walking or bicycling for travel of 130 min per week in females (*N* = 100, AUC 0.752, *P* < 0.001, sensitivity 0.710, specificity 0.742) and 165 min per week in males (*N* = 91, AUC 0.693, *P* = 0.003, sensitivity 0.689, specificity 0.667), and for vigorous-intensity activities at leisure of 113 min per week in females (*N* = 100, AUC 0.680, *P* = 0.004, sensitivity 0.884, specificity 0.452) and of 95 min per week in males (*N* = 91, AUC 0.765, *P* < 0.001, sensitivity 0.820, specificity 0.733). In our study population, the cut-off point next to the 150 min/week of total physical activity recommended by WHO was 162.5 min/week. For this value, we found a sensitivity of 0.236 and a specificity of 0.967.

### Depression Severity, Physical Activity, and Body Measures

Time with vigorous-intensity activities at work correlated significantly positively with HAMD scores at baseline. On the contrary, the time spent on walking or bicycling for travel correlated significantly negatively with BDI-II and HAMD scores. Consistent with that, we also found significant positive relationships between body fat mass and depression scores and significant negative relationships between body muscle mass and depression scores (**Table 2**). Physical activity and body measures were not significantly associated with a change in depression severity from study visit 1 to study visit 2. However, in sex-specific analyses, such effects were found in the group of female patients with an MDE. In this group, lower BMI, body weight, waist circumference, body fat mass, and visceral adipose tissue were associated with a stronger reduction on the HAMD score (**Supplemental Tables 3** and **4**). In line with the findings in the patients group, we also detected in the control group that HAMD scores correlated significantly positively with activities at work and significantly negatively with vigorous-intensity activities at leisure (**Supplemental Table 5**).

**Table 2:** Spearman correlations between physical activity, body measures, and depression severity in patients with a current MDE.

		BDI-II		HAMD	
		Study visit 1	Δ	Study visit 1	Δ
Total physical activity	*N*	127	118	127	118
	ρ	−0.147	0.153	0.040	0.028
	*P*	0.099	0.098	0.657	0.762
Vigorous-intensity activities at work	*N*	130	120	130	120
	ρ	0.069	0.055	0.247	−0.161
	*P*	0.434	0.554	**0.005**	0.079
Moderate-intensity activities at work	*N*	129	120	129	120
	ρ	−0.150	0.123	0.064	−0.040
	*P*	0.089	0.181	0.473	0.664
Time spent walking or bicycling for travel	*N*	130	120	130	120
	ρ	−0.207	0.158	-0.248	0.118
	*P*	**0.018**	0.086	**0.004**	0.199
Vigorous-intensity activities at leisure	*N*	130	120	130	120
	ρ	−0.074	−0.004	−0.084	−0.034
	*P*	0.401	0.966	0.340	0.710
Moderate-intensity activities at leisure	*N*	128	118	128	118
	ρ	−0.150	0.075	−0.081	0.083
	*P*	0.091	0.422	0.365	0.370
Sitting	*N*	130	120	130	120
	ρ	0.082	0.013	−0.115	0.004
	*P*	0.355	0.889	0.191	0.964
BMI	*N*	130	120	130	120
	ρ	0.042	0.127	0.141	0.057
	*P*	0.636	0.169	0.109	0.537
Body height	*N*	130	120	130	120
	ρ	−0.032	−0.141	−0.161	−0.053
	*P*	0.721	0.126	0.068	0.563
Body weight	*N*	130	120	130	120
	ρ	0.020	−0.021	0.032	−0.017
	*P*	0.824	0.819	0.715	0.853
Waist circumference	*N*	123	113	123	113
	ρ	0.033	−0.008	0.143	−0.004
	*P*	0.721	0.929	0.115	0.964
Body fat mass	*N*	127	117	127	117
	ρ	0.185	0.132	0.172	0.074
	*P*	**0.037**	0.157	0.053	0.425
Body muscle mass	*N*	127	117	127	117
	ρ	−0.225	−0.111	−0.198	−0.078
	*P*	**0.011**	0.234	**0.026**	0.401
Visceral adipose tissue	*N*	127	117	127	117
	ρ	−0.041	0.032	0.064	0.010
	*P*	0.646	0.730	0.475	0.912

P < 0.05 in bold print. Δ, absolute change of depression score between baseline (study visit 1) and follow-up (study visit 2). N, total number of individuals with data for these parameters.

### Physical Activity, Body Measures, and Lipid Serum Levels

Details on lipid serum levels and the use of lipid lowering agents in the sample analyzed here have already been published in Wagner et al. (18). In patients with a current MDE, vigorous-intensity activities at work correlated significantly positively with triglyceride levels and LDL/HDL ratios, and time spent walking or bicycling for travel correlated significantly negatively with triglycerides, total and LDL cholesterol, and LDL/HDL ratios (**Table 3**). The sex-specific analyses confirmed the significant negative correlations between time spent walking or bicycling for travel and triglycerides, total and LDL cholesterol, and LDL/HDL ratio in male patients with MDE (**Supplemental Table 6**). As expected, there were several significant associations between body measures and serum lipid profile (**Table 3** and **Supplemental Table 6**). In the group of healthy control subjects, moderate-intensity activities at leisure correlated significantly positively with triglycerides and total and LDL cholesterol in the total cohort and the female and male sub-cohorts. In line with the patients with a current MDE, body measures were significantly related to the serum lipid profile (**Supplemental Tables 7** and **8**).

**Table 3:** Spearman correlations between physical activity, body measures, and serum lipid profile at baseline in patients with current MDE.

		Triglycerides	Cholesterol			
			Total	HDL	LDL	LDL/HDL ratio
Total physical activity	*N*	127	127	127	127	127
	ρ	0.026	0.000	0.032	−0.004	−0.024
	*P*	0.774	0.999	0.724	0.967	0.790
Vigorous-intensity activities at work	*N*	130	130	130	130	130
	ρ	0.196	0.103	−0.104	0.144	0.191
	*P*	**0.025**	0.245	0.241	0.102	**0.030**
Moderate-intensity activities at work	*N*	129	129	129	129	129
	ρ	0.025	0.061	0.065	0.038	−0.011
	*P*	0.781	0.489	0.461	0.671	0.905
Time spent walking or bicycling for travel	*N*	130	130	130	130	130
	ρ	−0.207	−0.179	0.081	−0.211	−0.227
	*P*	**0.018**	**0.041**	0.362	**0.016**	**0.009**
Vigorous-intensity activities at leisure	*N*	130	130	130	130	130
	ρ	−0.012	0.004	−0.013	0.010	0.003
	*P*	0.891	0.966	0.886	0.911	0.969
Moderate-intensity activities at leisure	*N*	128	128	128	128	128
	ρ	−0.033	0.016	0.107	−0.007	−0.088
	*P*	0.711	0.861	0.230	0.940	0.324
Sitting	*N*	130	130	130	130	130
	ρ	−0.054	−0.137	−0.075	−0.148	−0.065
	*P*	0.543	0.120	0.395	0.093	0.466
BMI	*N*	130	130	130	130	130
	ρ	0.342	0.188	−0.347	0.310	0.460
	*P*	**<0.001**	**0.032**	**<0.001**	**<0.001**	**<0.001**
Body height	*N*	130	130	130	130	130
	ρ	0.263	−0.148	−0.433	−0.080	0.220
	*P*	**0.003**	0.093	**<0.001**	0.365	**0.012**
Body weight	*N*	130	130	130	130	130
	ρ	0.450	0.088	−0.523	0.223	0.505
	*P*	**<0.001**	0.320	**<0.001**	**0.011**	**<0.001**
Waist circumference	*N*	123	123	123	123	123
	ρ	0.468	0.161	−0.530	0.318	0.570
	*P*	**<0.001**	0.075	**<0.001**	**<0.001**	**<0.001**
Body fat mass	*N*	127	127	127	127	127
	ρ	0.019	0.167	0.117	0.174	0.068
	*P*	0.829	0.060	0.190	0.050	0.446
Body muscle mass	*N*	127	127	127	127	127
	ρ	0.059	−0.179	−0.283	−0.137	0.061
	*P*	0.508	**0.043**	**0.001**	0.123	0.498
Visceral adipose tissue	*N*	127	127	127	127	127
	ρ	0.440	0.269	−0.470	0.400	0.591
	*P*	**<0.001**	**0.002**	**<0.001**	**<0.001**	**<0.001**

N, total number of individuals with data for these parameters. P < 0.05 in bold print.

### Physical Activity and Body Measures

We found various significant correlations between physical activities and body measures (**Table 4**). In sex-specific analyses, these were found only in males but not in females (**Supplemental Tables 9** and **10**).

**Table 4:** Spearman correlations between physical activity and body measures.

		BMI	Body height	Body weight	Waist circum-ference	Body fat mass	Body muscle mass	Visceral adipose tissue
Patients with a current MDE								
Total physical activity	*N*	127	127	127	121	125	125	125
	ρ	−0.078	0.048	−0.040	−0.146	−0.210	0.211	−0.061
	*P*	0.380	0.595	0.657	0.109	**0.019**	**0.018**	0.499
Vigorous-intensity activities at work	*N*	130	130	130	123	127	127	127
	ρ	0.033	0.142	0.111	0.029	−0.199	0.240	0.082
	*P*	0.710	0.108	0.208	0.748	**0.025**	**0.007**	0.361
Moderate-intensity activities at work	*N*	129	129	129	122	126	126	126
	ρ	−0.030	0.044	0.006	−0.111	−0.121	0.122	−0.062
	*P*	0.733	0.617	0.947	0.224	0.177	0.175	0.493
Time spent walking or bicycling for travel	*N*	130	130	130	123	127	127	127
	ρ	−0.177	−0.015	−0.153	−0.215	−0.200	0.179	−0.111
	*P*	**0.044**	0.862	0.081	**0.017**	**0.025**	**0.044**	0.213
Vigorous-intensity activities at leisure	*N*	130	130	130	123	127	127	127
	ρ	0.011	0.029	0.013	−0.056	−0.070	0.105	−0.036
	*P*	0.904	0.745	0.885	0.538	0.431	0.242	0.686
Moderate-intensity activities at leisure	*N*	128	128	128	122	126	126	126
	ρ	−0.113	−0.128	−0.184	−0.097	0.014	−0.063	−0.070
	*P*	0.204	0.151	**0.038**	0.290	0.876	0.484	0.433
Sitting	*N*	130	130	130	123	127	127	127
	ρ	0.073	0.124	0.127	0.099	0.025	-0.001	0.053
	*P*	0.410	0.159	0.149	0.277	0.783	0.987	0.555
Healthy control subjects								
Total physical activity	*N*	61	61	61	57	59	59	59
	ρ	0.188	0.222	0.311	0.133	0.005	0.034	0.063
	*P*	0.147	0.085	**0.015**	0.325	0.968	0.800	0.638
Vigorous-intensity activities at work	*N*	61	61	61	57	59	59	59
	ρ	0.125	0.040	0.096	−0.012	0.171	−0.141	0.085
	*P*	0.337	0.757	0.463	0.929	0.194	0.286	0.520
Moderate-intensity activities at work	*N*	61	61	61	57	59	59	59
	ρ	0.006	0.001	0.016	−0.011	0.106	−0.103	0.038
	*P*	0.966	0.995	0.901	0.935	0.426	0.435	0.777
Time spent walking or bicycling for travel	*N*	61	61	61	57	59	59	59
	ρ	0.249	0.114	0.296	0.155	0.186	−0.169	0.122
	*P*	0.053	0.381	**0.021**	0.248	0.157	0.202	0.359
Vigorous-intensity activities at leisure	*N*	61	61	61	57	59	59	59
	ρ	−0.068	0.151	0.063	−0.138	−0.302	0.308	−0.183
	*P*	0.601	0.246	0.631	0.306	**0.020**	**0.017**	0.166
Moderate-intensity activities at leisure	*N*	61	61	61	57	59	59	59
	ρ	0.152	−0.056	0.053	0.007	0.064	−0.067	0.224
	*P*	0.242	0.666	0.683	0.958	0.628	0.613	0.088
Sitting	*N*	61	61	61	57	59	59	59
	ρ	−0.136	0.246	0.049	0.218	−0.265	0.314	−0.047
	*P*	0.296	0.056	0.708	0.103	**0.043**	**0.015**	0.721

N, total number of individuals with data for these parameters. P < 0.05 in bold print.

## Discussion

Physical activity is widely recommended to promote health and to protect against a broad range of diseases. In support of this, here we found a significantly higher amount of physical activity in healthy control subjects than in patients with a current MDE. Vigorous-intensity activities in leisure and time spent to travel to and from places by bike or on foot were especially more extensive in healthy control subjects. Interestingly, neither moderate-intensity activities at work nor at leisure varied significantly and consistently between patients with a current MDE and the controls in all three groups (total, female, and male groups). As the groups did not significantly differ with regard to time spent working (i.e., paid hours at work per week and paid months during the previous year), disability, unemployment, and sick leave are unlikely to account for this observation. In the total cohort and the male sub-cohort, there was no significant difference in our control parameter (daily time used for sitting) which might in part be due to recall bias in patients with a current MDE. The time spent sitting (480 min/day for patients with an MDE vs. 420 min/day for healthy control subjects) in our cohort agrees with data from Hagströmer et al. (22) who found 459 min of inactivity per day for adults in a huge population based study. Helgadottir et al. (28) found 546 min of sedentary behavior per day in persons with mild to moderate symptoms of depressive and/or anxiety disorders. They showed that depression severity is positively associated with time spent sedentary (28); we were not able to replicate this association.

We calculated ROC analysis and demonstrated that less than 465 min per week of total physical activity (sensitivity 0.543, specificity 0.852), less than 123 min per week spent walking or bicycling for travel (sensitivity 0.662, specificity 0.738), and less than 113 min per week with vigorous-intensity activities at leisure (sensitivity 0.854, specificity 0.590) optimally discriminates between patients with MDE and healthy control subjects. The WHO recommends at least 150 min of moderate physical activity per week to prevent NCDs (2). Our data suggest that longer times spent on physical activity might be more beneficial. The results of our cross-sectional study are not applicable to make any inferences about causality. It is also very important to mention the bidirectional relationship of physical activity and mental health. On the one hand, physical activity reduces the risk of future depression (29, 30). On the other hand, depression may also lead to inactivity and increased sedentary behavior (31). Using Mendelian randomization, Choi et al. (6) found evidence that physical activity demonstrated a potential causal relationship with depression. While the physical activities' impact on depressive symptoms seems not to be crucial (4), it appears to be more important in the prevention of mental illness. Although mainly leisure time associated physical activity has been shown to be helpful to prevent depression, office-based workplace physical activity interventions seem to be effective in improving well-being (32). Since office-based interventions seem to be applicable, they should be broader analyzed in future studies.

In patients with a current MDE, more time spent on traveling was associated with milder depression severity, while vigorous-intensity activities at work were associated with a higher HAMD score. Also, in the group of healthy control subjects, activities at work related to higher HAMD scores and vigorous activities at leisure were linked to lower HAMD scores. These results partly agree with those of Harvey at al. (5), who found on the one hand a protective impact of leisure-time physical activity in general but on the other hand a harming effect of physical activities at work. Here, the causality remains unclear as monotonous work or socioeconomic status might act as confounder.

In our study cohort, we found significant differences in the between-group comparisons. Healthy control subjects spent more time for total physical activity, walking or bicycling for travel, and vigorous-intensity activities at leisure. This agrees with Chekroud et al. (33) who found a significant reduction in mental health burden especially for popular sports, cycling, and aerobic and gym exercises compared with no exercise, preferably 30 to 60 min, three to five times per week, in a large cross-sectional study. In accordance with Cooney et al. (4), we also show that, once a patient has developed an MDE, physical activity seems not to be associated with the prospective course of depression *per se*, but does seem to be associated with milder depression severity.

**Figure 2:**
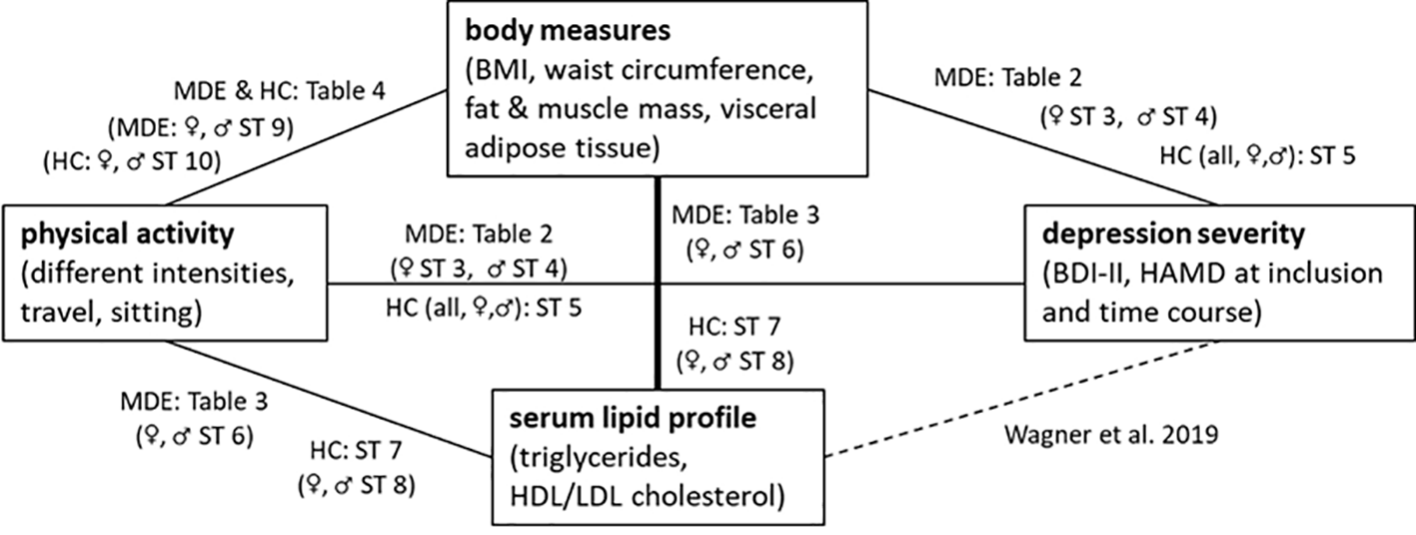
An overview of the correlations shown in the tables and supplemental tables (ST) for patients with a current MDE and healthy controls (HC).

As shown in **Figure 2**, we also explored the role of body measures and serum lipids in the relationship between physical activity and depression severity. The investigated body measurements did not significantly differ between the two groups, although some showed statistical trends. As expected, depressive patients tended to have higher body fat mass, higher visceral adipose tissue mass, and lower body muscle mass than the controls. These differences reached significance in the male sub-cohort. Altogether, this supports our results showing less physical activity in patients with a current MDE. The rather small number of overall study subjects might account for missing a significant effect. Moreover, the inconsistencies between the associations with self-reported physical activity and objective body measure might be due to recall bias in patients with a current MDE. Surprisingly, in the group of female patients with an MDE, we found that lower BMI, body weight, waist circumference, body fat mass, and visceral adipose tissue were associated with a stronger reduction in HAMD score in sex-specific analyses. Further studies for replication of this result are necessary.

Both physical activity and body measurements were strongly related to serum lipid levels. As described earlier, serum lipids, especially LDL cholesterol, are positively associated with depression and depression severity in our cohort (18). In this former study, we observed that treatment with antidepressants did not have a strong effect on the association of lipid levels with depression severity (18). In support of this Kuel et al. (34) did not find different cardiovascular risk profiles among depressed patients with and without antidepressant medication.

For patients with a current MDE, vigorous-intensity activities at work correlated positively with triglyceride levels and LDL/HDL ratios. Furthermore, time spent walking or bicycling for travel correlated negatively with triglyceride, total and LDL cholesterol, and LDL/HDL ratio. These associations suggest that serum lipids are involved in the relationship between physical activity and MDE. This also supports the assumption that subforms of physical activity differ in their impact on mental health with both helpful and harming effects. MDD, dyslipidemia, and insulin resistance are said to share immunoinflammatory alterations (35). Exercise training is potent to interrupt the obesity-induced inflammatory mechanisms (36) and can be utilized to improve cholesterol levels (37). As a result, physical activity might prevent depressive symptoms.

Body fat mass correlated positively and body muscle mass correlated negatively with depression severity, supporting the described associations between physical activity and depression. This agrees with Alshehri et al. (38), who detected a positive association between total body fat and depressive mood both in men and women. As expected, we found strong correlations between physical activity and body composition measurements. These support the validity of the self-reported physical activities. In line with our results, Choi et al. (6) found a protective effect against depression of objectively assessed but not self-reported physical activity.

Our study is limited by the associational study design, which does not allow causal conclusions. We analyzed a rather small study population and did not differentiate clinical characteristics of the episode like duration of the episodes or former episodes. We used self-reports of physical activity for our analyses without any direct objective measurements. Future studies might use pedometers. We also had only a small control population, which might account for distortion of the results. Moreover, we did not correct for multiple hypothesis testing, which might have resulted in some false positive findings. The strengths of our study include the systematic analyses of subforms of physical activity, the measurements of several body composition parameters, and the analyses of serum lipids.

In summary, our results support the recommendation that traveling by foot or by bike and vigorous-intensive physical activity at leisure should be widely promoted to prevent MDE.

## Data Availability Statement

The datasets are available on request. The raw data supporting the conclusions of this article will be made available by the authors, without undue reservation, to any qualified researcher.

## Ethics Statement

The studies involving human participants were reviewed and approved by Ethics Committee of the Medical Faculty of the Friedrich-Alexander University Erlangen-Nürnberg (ID 148_13 B). The patients/participants provided their written informed consent to participate in this study.

## Author Contributions

Conceived and designed the study: CZ, MW, TR-S, CM, JK, and BL. Performed the experiments: CZ, MW, CM, and BL. Analyzed the data and wrote the paper: CZ, MW, CM, and BL. Commented on the manuscript and provided intellectual input: TR-S and JK.

## Funding

This work was supported by grants of the German Federal Ministry of Education and Research (01EE1401C), the German Research Foundation (KO 947/13-3), and intramural grants from the University Hospital of the Friedrich-Alexander University Erlangen-Nürnberg (FAU). CM is an associated fellow of the research training group 2162 funded by the Deutsche Forschungsgemeinschaft (DFG, German Research Foundation) - 270949263/GRK2162. The funders had no role in the study design, data collection, analysis, decision to publish, or preparation of the manuscript.

## Conflict of Interest

The authors declare that the research was conducted in the absence of any commercial or financial relationships that could be construed as a potential conflict of interest.
