# Supplementary material for: Physical Activity and Body Composition Are Associated With Severity and Risk of Depression, and Serum Lipids
Source: Front Psychiatry. 2020 Jun 5;11:494. doi: 10.3389/fpsyt.2020.00494 (PMC7292005; doi:10.3389/fpsyt.2020.00494)
Supplement: Supplementary file 1 [file DataSheet_1.docx]

Supplementary Material

von Zimmermann C, Winkelmann M, Richter-Schmidinger T, Mühle C, Kornhuber J and Lenz B (2020) Physical activity and body composition are associated with severity and risk of depression, and serum lipids. Front. Psychiatry 11:494. doi: 10.3389/fpsyt.2020.00494

**Inclusion criteria**

- written informed consent
- age between 18 and 75 years
- for patients older than 65 years: more than 26 points at Mini-Mental State Examination
- BMI between 18.5 kg/m² and 35 kg/m²

Additional:

Patients with a current major depressive episode: age less than 60 years at the first depressive episode

Healthy control subjects: no further than already mentioned

**Exclusion criteria**

- acute or severe physical illness and autoimmune disorders (rheumatoid arthritis, lupus erythematodes, M. Addison, M. Cushing, cancer, multiple sclerosis, Parkinson’s disease, inflammatory bowel disease, psoriasis, chronic bronchitis, acute allergic rhinitis, type 1 diabetes, type 2 diabetes, stroke, acute cardiovascular disease, traumatic brain injury, current treatment of an infectious disease (like HIV, hepatitis c, tuberculosis), dementia, thyroid disease)
- use of anti-inflammatory drugs or corticosteroids in the last seven days
- pregnancy or breastfeeding
- legal care

Additional:

Patients with current major depressive disorder:

- mental illness
  - other than depressive episode, generalized anxiety disorder, social phobia, panic disorder, specific phobia, nicotine dependence
  - borderline disorder
  - psychotic symptoms
  - suicidal intentions or acute suicidal thoughts

Healthy control subjects: mental illness other than nicotine dependence

**Supplemental Table 1.** The female study cohort and group comparisons

|  | Patients with a current MDE | | | | Healthy control subjects | | | |  | Group comparisons | |
| --- | --- | --- | --- | --- | --- | --- | --- | --- | --- | --- | --- |
|  | *N* | % or Median | IQR | | *N* | % or Median | IQR | |  | χ^2^, df ^a^ or *U* ^b^ | *P* |
| Single (%) | 69 | 62 |  |  | 31 | 84 |  |  | ^a^ | 4.6, 1 | **0.031** |
| Married (%) | 69 | 42 |  |  | 31 | 48 |  |  | ^a^ | 0.4, 1 | 0.554 |
| Divorced (%) | 60 | 26 |  |  | 30 | 7 |  |  | ^a^ | 4.9, 1 | **0.027** |
| Age (years) | 69 | 45 | 32 | 54 | 31 | 47 | 32 | 60 | ^b^ | 986 | 0.531 |
| Sum of education years | 62 | 14 | 12 | 17 | 25 | 14 | 12 | 17 | ^b^ | 768 | 0.944 |
| Paid working hours per week | 63 | 15 | 0 | 39 | 25 | 15 | 0 | 30 | ^b^ | 718 | 0.512 |
| Paid working months during the previous year | 64 | 12 | 3 | 12 | 27 | 10 | 0 | 12 | ^b^ | 736 | 0.216 |
| Depression scores |  |  |  |  |  |  |  |  |  |  |  |
| BDI-II score at baseline | 69 | 30 | 24 | 37 | 31 | 1 | 0 | 4 | ^b^ | 4 | **<0.001** |
| BDI-II score at follow-up | 62 | 22 | 15 | 31 |  |  |  |  |  |  |  |
| HAMD score at baseline | 69 | 23 | 19 | 26 | 31 | 1 | 0 | 3 | ^b^ | 0 | **<0.001** |
| HAMD score at follow-up | 62 | 18 | 14 | 22 |  |  |  |  |  |  |  |
| Physical activity |  |  |  |  |  |  |  |  |  |  |  |
| Total physical activity (min/week) | 66 | 390 | 180 | 945 | 31 | 870 | 405 | 1620 | ^b^ | 649 | **0.004** |
| Vigorous-intensity activities at work (min/week) | 69 | 0 | 0 | 0 | 31 | 0 | 0 | 0 | ^b^ | 1011 | 0.376 |
| Moderate-intensity activities at work (min/week) | 68 | 0 | 0 | 435 | 31 | 0 | 0 | 450 | ^b^ | 1037 | 0.877 |
| Time spent walking or bicycling for travel (min/week) | 69 | 20 | 0 | 180 | 31 | 210 | 120 | 315 | ^b^ | 531 | **<0.001** |
| Vigorous-intensity activities at leisure (min/week) | 69 | 0 | 0 | 0 | 31 | 60 | 0 | 180 | ^b^ | 684 | **0.001** |
| Moderate-intensity activities at leisure (min/week) | 67 | 60 | 0 | 180 | 31 | 90 | 0 | 210 | ^b^ | 945 | 0.464 |
| Sitting (min/day) | 69 | 480 | 300 | 600 | 31 | 360 | 240 | 540 | ^b^ | 796 | **0.041** |
| Body measures |  |  |  |  |  |  |  |  |  |  |  |
| BMI (kg/m^2^) | 69 | 26 | 22 | 29 | 31 | 24 | 23 | 26 | ^b^ | 991 | 0.558 |
| Body height (cm) | 69 | 167 | 162 | 170 | 31 | 167 | 160 | 170 | ^b^ | 1050 | 0.881 |
| Body weight (kg) | 69 | 70 | 59 | 80 | 31 | 69 | 63 | 73 | ^b^ | 1018 | 0.701 |
| Waist circumference (cm) | 63 | 83 | 75 | 93 | 28 | 84 | 78 | 89 | ^b^ | 843 | 0.734 |
| Body fat mass (%) | 67 | 38 | 30 | 43 | 29 | 36 | 32 | 39 | ^b^ | 883 | 0.480 |
| Body muscle mass (%) | 67 | 26 | 25 | 29 | 29 | 27 | 26 | 29 | ^b^ | 863 | 0.384 |
| Visceral adipose tissue (%) | 67 | 7 | 4 | 9 | 29 | 6 | 5 | 8 | ^b^ | 887 | 0.495 |

MDE Major depressive episode, IQR interquartile range, BMI Body mass index. ^a^χ^2^ test, ^b^Mann-Whitney *U* test. *P*<0.05 in bold print. N total number of individuals with data for these parameters.

**Supplemental Table 2.** The male study cohort and group comparisons

|  | Patients with a current MDE | | | | Healthy control subjects | | | |  | Group comparisons | |
| --- | --- | --- | --- | --- | --- | --- | --- | --- | --- | --- | --- |
|  | *N* | % or Median | IQR | | *N* | % or Median | IQR | |  | χ^2^, df ^a^ or *U* ^b^ | *P* |
| Single (%) | 61 | 62 |  |  | 30 | 70 |  |  | ^a1^ | 0.5, 1 | 0.469 |
| Married (%) | 61 | 38 |  |  | 30 | 17 |  |  | ^a1^ | 4.2, 1 | **0.041** |
| Divorced (%) | 61 | 15 |  |  | 30 | 3 |  |  | ^a2^ | 2.7, 1 | 0.156 |
| Age (years) | 61 | 47 | 35 | 53 | 30 | 37 | 30 | 49 | ^b^ | 737 | 0.133 |
| Sum of education years | 53 | 15 | 13 | 17 | 26 | 17 | 14 | 18 | ^b^ | 552 | 0.150 |
| Paid working hours per week | 56 | 35 | 0 | 40 | 26 | 39 | 8 | 40 | ^b^ | 682 | 0.639 |
| Paid working months during the previous year | 56 | 12 | 2 | 12 | 26 | 12 | 6 | 12 | ^b^ | 685 | 0.623 |
| Depression scores |  |  |  |  |  |  |  |  |  |  |  |
| BDI-II score at baseline | 61 | 27 | 22 | 32 | 30 | 2 | 0 | 3 | ^b^ | 2 | **<0.001** |
| BDI-II score at follow-up | 58 | 17 | 13 | 23 |  |  |  |  |  |  |  |
| HAMD score at baseline | 61 | 21 | 20 | 24 | 30 | 0 | 0 | 1 | ^b^ | 0 | **<0.001** |
| HAMD score at follow-up | 58 | 15 | 11 | 20 |  |  |  |  |  |  |  |
| Physical activity |  |  |  |  |  |  |  |  |  |  |  |
| Total physical activity (min/week) | 61 | 480 | 150 | 1500 | 30 | 875 | 600 | 1890 | ^b^ | 648 | **0.024** |
| Vigorous-intensity activities at work (min/week) | 61 | 0 | 0 | 30 | 30 | 0 | 0 | 0 | ^b^ | 733 | **0.027** |
| Moderate-intensity activities at work (min/week) | 61 | 0 | 0 | 300 | 30 | 0 | 0 | 180 | ^b^ | 865 | 0.627 |
| Time spent walking or bicycling for travel (min/week) | 61 | 80 | 0 | 210 | 30 | 210 | 90 | 420 | ^b^ | 561 | **0.002** |
| Vigorous-intensity activities at leisure (min/week) | 61 | 0 | 0 | 30 | 30 | 180 | 60 | 270 | ^b^ | 430 | **<0.001** |
| Moderate-intensity activities at leisure (min/week) | 61 | 0 | 0 | 180 | 30 | 60 | 0 | 225 | ^b^ | 733 | 0.101 |
| Sitting (min/day) | 61 | 480 | 300 | 660 | 30 | 480 | 360 | 600 | ^b^ | 905 | 0.929 |
| Body measures |  |  |  |  |  |  |  |  |  |  |  |
| BMI (kg/m^2^) | 61 | 28 | 25 | 29 | 30 | 25 | 23 | 28 | ^b^ | 687 | 0.054 |
| Body height (cm) | 61 | 179 | 173 | 184 | 30 | 183 | 175 | 188 | ^b^ | 746 | 0.152 |
| Body weight (kg) | 61 | 86 | 80 | 96 | 30 | 89 | 75 | 97 | ^b^ | 853 | 0.601 |
| Waist circumference (cm) | 60 | 99 | 90 | 103 | 29 | 93 | 85 | 100 | ^b^ | 660 | 0.066 |
| Body fat mass (%) | 60 | 27 | 22 | 32 | 30 | 22 | 18 | 26 | ^b^ | 564 | **0.004** |
| Body muscle mass (%) | 60 | 33 | 31 | 36 | 30 | 36 | 34 | 40 | ^b^ | 541 | **0.002** |
| Visceral adipose tissue (%) | 60 | 11 | 9 | 13 | 30 | 8 | 6 | 12 | ^b^ | 624 | **0.018** |

MDE Major depressive episode, IQR interquartile range, BMI Body mass index. ^a1^χ^2^ test, ^a2^Fisher’s exact test because at least one cell with an expected count less than 5, ^b^Mann-Whitney *U* test. *P*<0.05 in bold print. N total number of individuals with data for these parameters.

**Supplemental Table 3.** Spearman correlations between physical activity, body measures, and depression severity in female patients with a current MDE

|  |  | BDI-II | | HAMD | |
| --- | --- | --- | --- | --- | --- |
|  |  | Study visit 1 | Δ | Study visit 1 | Δ |
| Total physical activity | *N* | 66 | 60 | 66 | 60 |
|  | ρ | -0.173 | 0.189 | -0.038 | 0.110 |
|  | *P* | 0.165 | 0.149 | 0.761 | 0.403 |
| Vigorous-intensity activities at work | *N* | 69 | 62 | 69 | 62 |
|  | ρ | 0.155 | 0.023 | 0.302 | -0.178 |
|  | *P* | 0.204 | 0.860 | **0.012** | 0.165 |
| Moderate-intensity activities at work | *N* | 68 | 62 | 68 | 62 |
|  | ρ | -0.213 | 0.156 | -0.013 | 0.062 |
|  | *P* | 0.082 | 0.225 | 0.916 | 0.632 |
| Time spent walking or bicycling for travel | *N* | 69 | 62 | 69 | 62 |
|  | ρ | -0.292 | 0.220 | -0.328 | 0.064 |
|  | *P* | **0.015** | 0.085 | **0.006** | 0.622 |
| Vigorous-intensity activities at leisure | *N* | 69 | 62 | 69 | 62 |
|  | ρ | 0.047 | 0.168 | 0.025 | -0.036 |
|  | *P* | 0.702 | 0.192 | 0.836 | 0.781 |
| Moderate-intensity activities at leisure | *N* | 67 | 60 | 67 | 60 |
|  | ρ | -0.266 | 0.142 | -0.074 | -0.020 |
|  | *P* | **0.030** | 0.278 | 0.550 | 0.878 |
| Sitting | *N* | 69 | 62 | 69 | 62 |
|  | ρ | 0.123 | -0.013 | -0.068 | 0.098 |
|  | *P* | 0.313 | 0.918 | 0.580 | 0.447 |
| BMI | *N* | 69 | 62 | 69 | 62 |
|  | ρ | 0.060 | 0.184 | 0.110 | 0.273 |
|  | *P* | 0.624 | 0.152 | 0.367 | **0.032** |
| Body height | *N* | 69 | 62 | 69 | 62 |
|  | ρ | 0.158 | -0.138 | -0.094 | 0.071 |
|  | *P* | 0.194 | 0.286 | 0.442 | 0.584 |
| Body weight | *N* | 69 | 62 | 69 | 62 |
|  | ρ | 0.120 | 0.131 | 0.102 | 0.287 |
|  | *P* | 0.327 | 0.311 | 0.406 | **0.024** |
| Waist circumference | *N* | 63 | 56 | 63 | 56 |
|  | ρ | 0.098 | 0.225 | 0.178 | 0.306 |
|  | *P* | 0.445 | 0.096 | 0.164 | **0.022** |
| Body fat mass | *N* | 67 | 60 | 67 | 60 |
|  | ρ | 0.105 | 0.186 | 0.149 | 0.291 |
|  | *P* | 0.396 | 0.155 | 0.228 | **0.024** |
| Body muscle mass | *N* | 67 | 60 | 67 | 60 |
|  | ρ | -0.090 | -0.146 | -0.182 | -0.206 |
|  | *P* | 0.466 | 0.266 | 0.141 | 0.115 |
| Visceral adipose tissue | *N* | 67 | 60 | 67 | 60 |
|  | ρ | 0.048 | 0.187 | 0.056 | 0.284 |
|  | *P* | 0.702 | 0.152 | 0.652 | **0.028** |

*P*<0.05 in bold print. Δ absolute change of depression score between baseline (study visit 1) and follow-up (study visit 2). N total number of individuals with data for these parameters.

**Supplemental Table 4.** Spearman correlations between physical activity, body measures, and depression severity in male patients with a current MDE

|  |  | BDI-II | | HAMD | |
| --- | --- | --- | --- | --- | --- |
|  |  | Study visit 1 | Δ | Study visit 1 | Δ |
| Total physical activity | *N* | 61 | 58 | 61 | 58 |
|  | ρ | -0.110 | 0.165 | 0.156 | -0.035 |
|  | *P* | 0.400 | 0.217 | 0.230 | 0.794 |
| Vigorous-intensity activities at work | *N* | 61 | 58 | 61 | 58 |
|  | ρ | 0.092 | 0.139 | 0.308 | -0.116 |
|  | *P* | 0.482 | 0.298 | **0.016** | 0.385 |
| Moderate-intensity activities at work | *N* | 61 | 58 | 61 | 58 |
|  | ρ | -0.076 | 0.082 | 0.187 | -0.148 |
|  | *P* | 0.561 | 0.542 | 0.148 | 0.267 |
| Time spent walking or bicycling for travel | *N* | 61 | 58 | 61 | 58 |
|  | ρ | -0.065 | 0.112 | -0.129 | 0.193 |
|  | *P* | 0.619 | 0.402 | 0.322 | 0.147 |
| Vigorous-intensity activities at leisure | *N* | 61 | 58 | 61 | 58 |
|  | ρ | -0.215 | -0.156 | -0.194 | -0.032 |
|  | *P* | 0.096 | 0.243 | 0.134 | 0.809 |
| Moderate-intensity activities at leisure | *N* | 61 | 58 | 61 | 58 |
|  | ρ | -0.065 | -0.037 | -0.116 | 0.141 |
|  | *P* | 0.619 | 0.785 | 0.372 | 0.291 |
| Sitting | *N* | 61 | 58 | 61 | 58 |
|  | ρ | 0.063 | 0.089 | -0.147 | -0.087 |
|  | *P* | 0.631 | 0.504 | 0.259 | 0.518 |
| BMI | *N* | 61 | 58 | 61 | 58 |
|  | ρ | 0.075 | 0.132 | 0.235 | -0.200 |
|  | *P* | 0.564 | 0.324 | 0.068 | 0.133 |
| Body height | *N* | 61 | 58 | 61 | 58 |
|  | ρ | 0.170 | -0.151 | -0.179 | 0.027 |
|  | *P* | 0.190 | 0.259 | 0.168 | 0.840 |
| Body weight | *N* | 61 | 58 | 61 | 58 |
|  | ρ | 0.127 | 0.001 | 0.062 | -0.199 |
|  | *P* | 0.328 | 0.994 | 0.633 | 0.135 |
| Waist circumference | *N* | 60 | 57 | 60 | 57 |
|  | ρ | 0.058 | -0.047 | 0.114 | -0.116 |
|  | *P* | 0.660 | 0.731 | 0.386 | 0.391 |
| Body fat mass | *N* | 60 | 57 | 60 | 57 |
|  | ρ | 0.138 | -0.035 | 0.143 | -0.262 |
|  | *P* | 0.293 | 0.795 | 0.274 | **0.049** |
| Body muscle mass | *N* | 60 | 57 | 60 | 57 |
|  | ρ | -0.184 | 0.096 | -0.140 | 0.214 |
|  | *P* | 0.159 | 0.477 | 0.286 | 0.109 |
| Visceral adipose tissue | *N* | 60 | 57 | 60 | 57 |
|  | ρ | 0.122 | 0.085 | 0.240 | -0.097 |
|  | *P* | 0.352 | 0.527 | 0.065 | 0.474 |

*P*<0.05 in bold print. Δ absolute change of depression score between baseline (study visit 1) and follow-up (study visit 2). N total number of individuals with data for these parameters.

**Supplemental Table 5.** Spearman correlations between physical activity, body measures, and depression severity in healthy control subjects (total cohort, females, and males)

|  |  | Total cohort | | Females | | Males | |
| --- | --- | --- | --- | --- | --- | --- | --- |
|  |  | BDI-II | HAMD | BDI-II | HAMD | BDI-II | HAMD |
| Total physical activity | *N* | 61 | 61 | 31 | 31 | 30 | 30 |
|  | ρ | 0.004 | 0.042 | 0.202 | 0.069 | -0.174 | 0.107 |
|  | *P* | 0.977 | 0.751 | 0.275 | 0.714 | 0.358 | 0.575 |
| Vigorous-intensity activities at work | *N* | 61 | 61 | 31 | 31 | 30 | 30 |
|  | ρ | 0.065 | 0.344 | 0.233 | 0.338 | -0.187 | 0.337 |
|  | *P* | 0.619 | **0.007** | 0.207 | 0.063 | 0.322 | 0.069 |
| Moderate-intensity activities at work | *N* | 61 | 61 | 31 | 31 | 30 | 30 |
|  | ρ | 0.070 | 0.296 | 0.014 | 0.361 | 0.079 | 0.126 |
|  | *P* | 0.591 | **0.020** | 0.940 | **0.046** | 0.677 | 0.508 |
| Time spent walking or bicycling for travel | *N* | 61 | 61 | 31 | 31 | 30 | 30 |
|  | ρ | -0.050 | -0.007 | 0.097 | 0.036 | -0.182 | -0.006 |
|  | *P* | 0.703 | 0.956 | 0.603 | 0.849 | 0.336 | 0.974 |
| Vigorous-intensity activities at leisure | *N* | 61 | 61 | 31 | 31 | 30 | 30 |
|  | ρ | -0.127 | -0.270 | 0.023 | -0.333 | -0.294 | -0.071 |
|  | *P* | 0.331 | **0.035** | 0.901 | 0.068 | 0.115 | 0.708 |
| Moderate-intensity activities at leisure | *N* | 61 | 61 | 31 | 31 | 30 | 30 |
|  | ρ | 0.108 | -0.092 | 0.032 | -0.039 | 0.230 | -0.147 |
|  | *P* | 0.408 | 0.481 | 0.863 | 0.836 | 0.222 | 0.440 |
| Sitting | *N* | 61 | 61 | 31 | 31 | 30 | 30 |
|  | ρ | -0.096 | -0.140 | -0.148 | -0.014 | -0.063 | -0.113 |
|  | *P* | 0.462 | 0.283 | 0.428 | 0.942 | 0.741 | 0.550 |
| BMI | *N* | 61 | 61 | 31 | 31 | 30 | 30 |
|  | ρ | -0.008 | -0.135 | 0.078 | -0.283 | -0.044 | 0.074 |
|  | *P* | 0.948 | 0.301 | 0.677 | 0.124 | 0.816 | 0.697 |
| Body height | *N* | 61 | 61 | 31 | 31 | 30 | 30 |
|  | ρ | -0.005 | -0.085 | 0.054 | 0.202 | 0.081 | 0.065 |
|  | *P* | 0.970 | 0.517 | 0.774 | 0.277 | 0.671 | 0.732 |
| Body weight | *N* | 61 | 61 | 31 | 31 | 30 | 30 |
|  | ρ | -0.043 | -0.144 | 0.048 | -0.184 | -0.022 | 0.169 |
|  | *P* | 0.744 | 0.269 | 0.797 | 0.321 | 0.909 | 0.371 |
| Waist circumference | *N* | 57 | 57 | 28 | 28 | 29 | 29 |
|  | ρ | 0.091 | -0.070 | 0.087 | -0.045 | 0.252 | 0.287 |
|  | *P* | 0.500 | 0.604 | 0.661 | 0.821 | 0.187 | 0.132 |
| Body fat mass | *N* | 59 | 59 | 29 | 29 | 30 | 30 |
|  | ρ | 0.022 | 0.197 | 0.016 | -0.093 | 0.024 | 0.135 |
|  | *P* | 0.868 | 0.135 | 0.933 | 0.632 | 0.899 | 0.476 |
| Body muscle mass | *N* | 59 | 59 | 29 | 29 | 30 | 30 |
|  | ρ | -0.044 | -0.254 | -0.002 | 0.009 | -0.117 | -0.217 |
|  | *P* | 0.741 | 0.052 | 0.993 | 0.963 | 0.538 | 0.249 |
| Visceral adipose tissue | *N* | 59 | 59 | 29 | 29 | 30 | 30 |
|  | ρ | -0.055 | -0.062 | 0.011 | 0.055 | -0.017 | 0.054 |
|  | *P* | 0.678 | 0.639 | 0.953 | 0.775 | 0.928 | 0.779 |

*P*<0.05 in bold print. N total number of individuals with data for these parameters.

**Supplemental Table 6.** Spearman correlations between physical activity, body measures, and serum lipid profile at baseline in female and male patients with a current MDE

|  |  | Females | | | | | Males | | | | |
| --- | --- | --- | --- | --- | --- | --- | --- | --- | --- | --- | --- |
|  |  | Trigly-  cerides | Cholesterol | | | | Trigly-  cerides | Cholesterol | | | |
|  |  |  | Total | HDL | LDL | LDL/  HDL  ratio |  | Total | HDL | LDL | LDL/  HDL  ratio |
| Total physical activity | *N* | 66 | 66 | 66 | 66 | 66 | 61 | 61 | 61 | 61 | 61 |
|  | ρ | -0.119 | 0.019 | 0.029 | -0.009 | 0.000 | 0.117 | -0.035 | 0.111 | -0.044 | -0.104 |
|  | *P* | 0.341 | 0.882 | 0.816 | 0.945 | 0.997 | 0.368 | 0.788 | 0.396 | 0.738 | 0.424 |
| Vigorous-intensity activities at work | *N* | 69 | 69 | 69 | 69 | 69 | 61 | 61 | 61 | 61 | 61 |
|  | ρ | 0.010 | -0.031 | 0.012 | -0.027 | -0.002 | 0.192 | 0.180 | 0.025 | 0.203 | 0.192 |
|  | *P* | 0.932 | 0.802 | 0.921 | 0.829 | 0.986 | 0.139 | 0.164 | 0.850 | 0.118 | 0.138 |
| Moderate-intensity activities at work | *N* | 68 | 68 | 68 | 68 | 68 | 61 | 61 | 61 | 61 | 61 |
|  | ρ | -0.044 | 0.100 | 0.048 | 0.064 | 0.016 | 0.130 | 0.005 | 0.087 | -0.013 | -0.025 |
|  | *P* | 0.719 | 0.416 | 0.700 | 0.605 | 0.899 | 0.318 | 0.970 | 0.503 | 0.920 | 0.847 |
| Time spent walking or bicycling for travel | *N* | 69 | 69 | 69 | 69 | 69 | 61 | 61 | 61 | 61 | 61 |
|  | ρ | -0.183 | -0.038 | 0.113 | -0.093 | -0.127 | -0.300 | -0.327 | 0.168 | -0.359 | -0.453 |
|  | *P* | 0.133 | 0.755 | 0.356 | 0.447 | 0.300 | **0.019** | **0.010** | 0.195 | **0.004** | **<0.001** |
| Vigorous-intensity activities at leisure | *N* | 69 | 69 | 69 | 69 | 69 | 61 | 61 | 61 | 61 | 61 |
|  | ρ | 0.030 | 0.072 | -0.030 | 0.099 | 0.102 | -0.098 | -0.076 | 0.093 | -0.097 | -0.212 |
|  | *P* | 0.810 | 0.556 | 0.806 | 0.419 | 0.402 | 0.451 | 0.561 | 0.478 | 0.458 | 0.101 |
| Moderate-intensity activities at leisure | *N* | 67 | 67 | 67 | 67 | 67 | 61 | 61 | 61 | 61 | 61 |
|  | ρ | -0.071 | 0.075 | 0.101 | 0.039 | -0.012 | 0.128 | -0.029 | 0.006 | -0.044 | -0.069 |
|  | *P* | 0.570 | 0.548 | 0.415 | 0.755 | 0.923 | 0.326 | 0.827 | 0.961 | 0.736 | 0.595 |
| Sitting | *N* | 69 | 69 | 69 | 69 | 69 | 61 | 61 | 61 | 61 | 61 |
|  | ρ | -0.001 | -0.136 | -0.095 | -0.161 | -0.059 | -0.161 | -0.139 | 0.028 | -0.167 | -0.174 |
|  | *P* | 0.994 | 0.264 | 0.437 | 0.187 | 0.632 | 0.215 | 0.285 | 0.831 | 0.199 | 0.180 |
| BMI | *N* | 69 | 69 | 69 | 69 | 69 | 61 | 61 | 61 | 61 | 61 |
|  | ρ | 0.235 | 0.296 | -0.283 | 0.401 | 0.460 | 0.344 | 0.103 | -0.359 | 0.155 | 0.345 |
|  | *P* | 0.052 | **0.014** | **0.018** | **0.001** | **<0.001** | **0.007** | 0.429 | **0.004** | 0.234 | **0.006** |
| Body height | *N* | 69 | 69 | 69 | 69 | 69 | 61 | 61 | 61 | 61 | 61 |
|  | ρ | -0.172 | -0.279 | 0.061 | -0.338 | -0.265 | 0.166 | -0.238 | -0.193 | -0.196 | -0.005 |
|  | *P* | 0.158 | **0.020** | 0.617 | **0.005** | **0.028** | 0.200 | 0.065 | 0.136 | 0.130 | 0.969 |
| Body weight | *N* | 69 | 69 | 69 | 69 | 69 | 61 | 61 | 61 | 61 | 61 |
|  | ρ | 0.212 | 0.204 | -0.236 | 0.272 | 0.347 | 0.413 | 0.015 | -0.427 | 0.081 | 0.346 |
|  | *P* | 0.081 | 0.092 | 0.051 | **0.024** | **0.003** | **0.001** | 0.908 | **0.001** | 0.533 | **0.006** |
| Waist circumference | *N* | 63 | 63 | 63 | 63 | 63 | 60 | 60 | 60 | 60 | 60 |
|  | ρ | 0.258 | 0.256 | -0.336 | 0.378 | 0.466 | 0.426 | 0.169 | -0.426 | 0.217 | 0.463 |
|  | *P* | **0.041** | **0.043** | **0.007** | **0.002** | **<0.001** | **0.001** | 0.197 | **0.001** | 0.096 | **<0.001** |
| Body fat mass | *N* | 67 | 67 | 67 | 67 | 67 | 60 | 60 | 60 | 60 | 60 |
|  | ρ | 0.254 | 0.319 | -0.259 | 0.412 | 0.465 | 0.329 | 0.051 | -0.391 | 0.104 | 0.310 |
|  | *P* | **0.038** | **0.009** | **0.035** | **0.001** | **<0.001** | **0.010** | 0.698 | **0.002** | 0.428 | **0.016** |
| Body muscle mass | *N* | 67 | 67 | 67 | 67 | 67 | 60 | 60 | 60 | 60 | 60 |
|  | ρ | -0.305 | -0.398 | 0.186 | -0.459 | -0.469 | -0.317 | -0.138 | 0.336 | -0.180 | -0.337 |
|  | *P* | **0.012** | **0.001** | 0.132 | **<0.001** | **<0.001** | **0.013** | 0.292 | **0.009** | 0.168 | **0.008** |
| Visceral adipose tissue | *N* | 67 | 67 | 67 | 67 | 67 | 60 | 60 | 60 | 60 | 60 |
|  | ρ | 0.254 | 0.404 | -0.219 | 0.488 | 0.492 | 0.370 | 0.242 | -0.303 | 0.261 | 0.382 |
|  | *P* | **0.038** | **0.001** | 0.075 | **<0.001** | **<0.001** | **0.004** | 0.062 | **0.019** | **0.044** | **0.003** |

*P*<0.05 in bold print. N total number of individuals with data for these parameters.

**Supplemental Table 7.** Spearman correlations between physical activity, body measures, and serum lipid profile in healthy control subjects

|  |  | Trigly-  cerides | Cholesterol | | | |
| --- | --- | --- | --- | --- | --- | --- |
|  |  |  | Total | HDL | LDL | LDL/HDL  ratio |
| Total physical activity | *N* | 61 | 61 | 61 | 61 | 61 |
|  | ρ | 0.035 | 0.111 | 0.007 | 0.126 | 0.164 |
|  | *P* | 0.791 | 0.393 | 0.957 | 0.334 | 0.207 |
| Vigorous-intensity activities at work | *N* | 61 | 61 | 61 | 61 | 61 |
|  | ρ | 0.030 | -0.063 | -0.058 | 0.005 | 0.125 |
|  | *P* | 0.817 | 0.631 | 0.655 | 0.971 | 0.336 |
| Moderate-intensity activities at work | *N* | 61 | 61 | 61 | 61 | 61 |
|  | ρ | -0.097 | 0.024 | 0.241 | -0.028 | -0.127 |
|  | *P* | 0.455 | 0.852 | 0.062 | 0.832 | 0.328 |
| Time spent walking or bicycling for travel | *N* | 61 | 61 | 61 | 61 | 61 |
|  | ρ | 0.207 | 0.188 | -0.027 | 0.188 | 0.172 |
|  | *P* | 0.109 | 0.146 | 0.837 | 0.147 | 0.184 |
| Vigorous-intensity activities at leisure | *N* | 61 | 61 | 61 | 61 | 61 |
|  | ρ | -0.103 | -0.152 | -0.208 | -0.097 | 0.076 |
|  | *P* | 0.429 | 0.242 | 0.107 | 0.457 | 0.561 |
| Moderate-intensity activities at leisure | *N* | 61 | 61 | 61 | 61 | 61 |
|  | ρ | 0.271 | 0.445 | 0.136 | 0.407 | 0.240 |
|  | *P* | **0.035** | **<0.001** | 0.297 | **0.001** | 0.063 |
| Sitting | *N* | 61 | 61 | 61 | 61 | 61 |
|  | ρ | -0.085 | -0.089 | -0.136 | -0.089 | -0.054 |
|  | *P* | 0.516 | 0.497 | 0.295 | 0.497 | 0.679 |
| BMI | *N* | 61 | 61 | 61 | 61 | 61 |
|  | ρ | 0.398 | 0.274 | -0.287 | 0.366 | 0.548 |
|  | *P* | **0.001** | **0.033** | **0.025** | **0.004** | **<0.001** |
| Body height | *N* | 61 | 61 | 61 | 61 | 61 |
|  | ρ | -0.003 | -0.100 | -0.259 | -0.055 | 0.192 |
|  | *P* | 0.983 | 0.443 | **0.044** | 0.676 | 0.139 |
| Body weight | *N* | 61 | 61 | 61 | 61 | 61 |
|  | ρ | 0.294 | 0.158 | -0.381 | 0.250 | 0.539 |
|  | *P* | **0.021** | 0.224 | **0.002** | 0.052 | **<0.001** |
| Waist circumference | *N* | 57 | 57 | 57 | 57 | 57 |
|  | ρ | 0.367 | 0.275 | -0.337 | 0.351 | 0.540 |
|  | *P* | **0.005** | **0.039** | **0.010** | **0.007** | **<0.001** |
| Body fat mass | *N* | 59 | 59 | 59 | 59 | 59 |
|  | ρ | 0.257 | 0.310 | 0.262 | 0.284 | 0.109 |
|  | *P* | **0.049** | **0.017** | **0.045** | **0.029** | 0.412 |
| Body muscle mass | *N* | 59 | 59 | 59 | 59 | 59 |
|  | ρ | -0.233 | -0.329 | -0.351 | -0.285 | -0.044 |
|  | *P* | 0.076 | **0.011** | **0.006** | **0.029** | 0.743 |
| Visceral adipose tissue | *N* | 59 | 59 | 59 | 59 | 59 |
|  | ρ | 0.508 | 0.348 | -0.372 | 0.452 | 0.626 |
|  | *P* | **<0.001** | **0.007** | **0.004** | **<0.001** | **<0.001** |

*P*<0.05 in bold print. N total number of individuals with data for these parameters.

**Supplemental Table 8.** Spearman correlations between physical activity, body measures, and serum lipid profile at baseline in female and male healthy control subjects

|  |  | Females | | | | | Males | | | | |
| --- | --- | --- | --- | --- | --- | --- | --- | --- | --- | --- | --- |
|  |  | Trigly-  cerides | Cholesterol | | | | Trigly-  cerides | Cholesterol | | | |
|  |  |  | Total | HDL | LDL | LDL/  HDL  ratio |  | Total | HDL | LDL | LDL/  HDL  ratio |
| Total physical activity | *N* | 31 | 31 | 31 | 31 | 31 | 30 | 30 | 30 | 30 | 30 |
|  | ρ | -0.155 | 0.055 | 0.126 | 0.005 | -0.093 | 0.174 | 0.209 | -0.122 | 0.294 | 0.344 |
|  | *P* | 0.405 | 0.767 | 0.498 | 0.978 | 0.619 | 0.357 | 0.267 | 0.521 | 0.115 | 0.063 |
| Vigorous-intensity activities at work | *N* | 31 | 31 | 31 | 31 | 31 | 30 | 30 | 30 | 30 | 30 |
|  | ρ | -0.055 | 0.061 | -0.041 | 0.081 | 0.113 | 0.095 | -0.246 | -0.403 | -0.091 | 0.280 |
|  | *P* | 0.770 | 0.743 | 0.825 | 0.665 | 0.546 | 0.617 | 0.189 | **0.027** | 0.632 | 0.134 |
| Moderate-intensity activities at work | *N* | 31 | 31 | 31 | 31 | 31 | 30 | 30 | 30 | 30 | 30 |
|  | ρ | -0.271 | -0.074 | 0.354 | -0.202 | -0.414 | 0.054 | 0.126 | 0.074 | 0.206 | 0.155 |
|  | *P* | 0.141 | 0.691 | 0.051 | 0.276 | **0.021** | 0.777 | 0.507 | 0.696 | 0.274 | 0.412 |
| Time spent walking or bicycling for travel | *N* | 31 | 31 | 31 | 31 | 31 | 30 | 30 | 30 | 30 | 30 |
|  | ρ | 0.130 | -0.054 | -0.065 | -0.054 | -0.053 | 0.282 | 0.404 | -0.021 | 0.411 | 0.309 |
|  | *P* | 0.487 | 0.771 | 0.730 | 0.774 | 0.775 | 0.131 | **0.027** | 0.913 | **0.024** | 0.097 |
| Vigorous-intensity activities at leisure | *N* | 31 | 31 | 31 | 31 | 31 | 30 | 30 | 30 | 30 | 30 |
|  | ρ | 0.012 | -0.058 | -0.260 | 0.002 | 0.147 | -0.261 | -0.258 | -0.007 | -0.257 | -0.182 |
|  | *P* | 0.950 | 0.758 | 0.158 | 0.990 | 0.430 | 0.164 | 0.168 | 0.973 | 0.171 | 0.336 |
| Moderate-intensity activities at leisure | *N* | 31 | 31 | 31 | 31 | 31 | 30 | 30 | 30 | 30 | 30 |
|  | ρ | 0.251 | 0.505 | 0.102 | 0.455 | 0.288 | 0.306 | 0.436 | 0.124 | 0.405 | 0.234 |
|  | *P* | 0.174 | **0.004** | 0.587 | **0.010** | 0.116 | 0.100 | **0.016** | 0.513 | **0.027** | 0.214 |
| Sitting | *N* | 31 | 31 | 31 | 31 | 31 | 30 | 30 | 30 | 30 | 30 |
|  | ρ | -0.101 | -0.226 | 0.124 | -0.254 | -0.254 | -0.075 | 0.186 | -0.045 | 0.160 | 0.075 |
|  | *P* | 0.587 | 0.221 | 0.508 | 0.168 | 0.167 | 0.695 | 0.325 | 0.814 | 0.399 | 0.694 |
| BMI | *N* | 31 | 31 | 31 | 31 | 31 | 30 | 30 | 30 | 30 | 30 |
|  | ρ | 0.280 | 0.098 | -0.308 | 0.186 | 0.374 | 0.383 | 0.439 | -0.238 | 0.541 | 0.604 |
|  | *P* | 0.127 | 0.601 | 0.092 | 0.316 | **0.038** | **0.037** | **0.015** | 0.206 | **0.002** | **<0.001** |
| Body height | *N* | 31 | 31 | 31 | 31 | 31 | 30 | 30 | 30 | 30 | 30 |
|  | ρ | -0.334 | -0.170 | 0.092 | -0.227 | -0.178 | 0.084 | 0.217 | 0.100 | 0.192 | 0.183 |
|  | *P* | 0.067 | 0.361 | 0.623 | 0.220 | 0.339 | 0.659 | 0.249 | 0.597 | 0.308 | 0.333 |
| Body weight | *N* | 31 | 31 | 31 | 31 | 31 | 30 | 30 | 30 | 30 | 30 |
|  | ρ | 0.105 | 0.072 | -0.224 | 0.117 | 0.274 | 0.482 | 0.537 | -0.152 | 0.606 | 0.672 |
|  | *P* | 0.573 | 0.701 | 0.226 | 0.530 | 0.136 | **0.007** | **0.002** | 0.422 | **<0.001** | **<0.001** |
| Waist circumference | *N* | 28 | 28 | 28 | 28 | 28 | 29 | 29 | 29 | 29 | 29 |
|  | ρ | 0.191 | 0.183 | -0.186 | 0.226 | 0.374 | 0.583 | 0.558 | -0.119 | 0.591 | 0.593 |
|  | *P* | 0.330 | 0.351 | 0.345 | 0.247 | 0.050 | **0.001** | **0.002** | 0.540 | **0.001** | **0.001** |
| Body fat mass | *N* | 29 | 29 | 29 | 29 | 29 | 30 | 30 | 30 | 30 | 30 |
|  | ρ | 0.367 | 0.098 | -0.267 | 0.197 | 0.337 | 0.573 | 0.536 | -0.121 | 0.583 | 0.606 |
|  | *P* | 0.050 | 0.613 | 0.162 | 0.306 | 0.074 | **0.001** | **0.002** | 0.524 | **0.001** | **<0.001** |
| Body muscle mass | *N* | 29 | 29 | 29 | 29 | 29 | 30 | 30 | 30 | 30 | 30 |
|  | ρ | -0.419 | -0.129 | 0.245 | -0.258 | -0.339 | -0.643 | -0.647 | 0.020 | -0.658 | -0.601 |
|  | *P* | **0.024** | 0.503 | 0.201 | 0.176 | 0.072 | **<0.001** | **<0.001** | 0.918 | **<0.001** | **<0.001** |
| Visceral adipose tissue | *N* | 29 | 29 | 29 | 29 | 29 | 30 | 30 | 30 | 30 | 30 |
|  | ρ | 0.444 | 0.345 | -0.189 | 0.428 | 0.468 | 0.575 | 0.532 | -0.236 | 0.610 | 0.657 |
|  | *P* | **0.016** | 0.067 | 0.326 | **0.020** | **0.010** | **0.001** | **0.002** | 0.209 | **<0.001** | **<0.001** |

*P*<0.05 in bold print. N total number of individuals with data for these parameters.

**Supplemental Table 9.** Spearman correlations between physical activity and body measures in female and male patients with a current MDE

|  |  | BMI | Body height | Body weight | Waist circum-ference | Body fat mass | Body muscle mass | Visceral adipose tissue |
| --- | --- | --- | --- | --- | --- | --- | --- | --- |
| Females |  |  |  |  |  |  |  |  |
| Total physical activity | *N* | 66 | 66 | 66 | 61 | 65 | 65 | 65 |
|  | ρ | -0.033 | 0.060 | 0.013 | -0.102 | -0.109 | 0.097 | -0.053 |
|  | *P* | 0.792 | 0.633 | 0.916 | 0.435 | 0.389 | 0.440 | 0.675 |
| Vigorous-intensity activities at work | *N* | 69 | 69 | 69 | 63 | 67 | 67 | 67 |
|  | ρ | -0.026 | 0.062 | 0.002 | -0.150 | -0.119 | 0.089 | -0.061 |
|  | *P* | 0.834 | 0.611 | 0.990 | 0.242 | 0.339 | 0.476 | 0.622 |
| Moderate-intensity activities at work | *N* | 68 | 68 | 68 | 62 | 66 | 66 | 66 |
|  | ρ | 0.035 | 0.150 | 0.102 | -0.030 | -0.083 | 0.090 | 0.035 |
|  | *P* | 0.775 | 0.223 | 0.406 | 0.814 | 0.506 | 0.474 | 0.779 |
| Time spent walking or bicycling for travel | *N* | 69 | 69 | 69 | 63 | 67 | 67 | 67 |
|  | ρ | -0.161 | -0.016 | -0.169 | -0.168 | -0.133 | 0.052 | -0.119 |
|  | *P* | 0.186 | 0.895 | 0.166 | 0.187 | 0.285 | 0.678 | 0.339 |
| Vigorous-intensity activities at leisure | *N* | 69 | 69 | 69 | 63 | 67 | 67 | 67 |
|  | ρ | 0.047 | -0.030 | 0.061 | 0.017 | 0.030 | 0.054 | -0.044 |
|  | *P* | 0.704 | 0.810 | 0.616 | 0.897 | 0.810 | 0.666 | 0.723 |
| Moderate-intensity activities at leisure | *N* | 67 | 67 | 67 | 62 | 66 | 66 | 66 |
|  | ρ | -0.085 | -0.194 | -0.154 | -0.096 | -0.054 | 0.048 | -0.038 |
|  | *P* | 0.494 | 0.117 | 0.213 | 0.457 | 0.664 | 0.704 | 0.763 |
| Sitting | *N* | 69 | 69 | 69 | 63 | 67 | 67 | 67 |
|  | ρ | 0.059 | 0.133 | 0.110 | 0.089 | 0.117 | -0.112 | 0.035 |
|  | *P* | 0.628 | 0.275 | 0.366 | 0.489 | 0.347 | 0.365 | 0.781 |
| Males |  |  |  |  |  |  |  |  |
| Total physical activity | *N* | 61 | 61 | 61 | 60 | 60 | 60 | 60 |
|  | ρ | -0.153 | -0.119 | -0.201 | -0.216 | -0.289 | 0.332 | -0.146 |
|  | *P* | 0.239 | 0.360 | 0.120 | 0.098 | **0.025** | **0.009** | 0.264 |
| Vigorous-intensity activities at work | *N* | 61 | 61 | 61 | 60 | 60 | 60 | 60 |
|  | ρ | -0.018 | -0.136 | -0.097 | -0.085 | -0.055 | 0.103 | -0.048 |
|  | *P* | 0.892 | 0.295 | 0.457 | 0.521 | 0.679 | 0.433 | 0.718 |
| Moderate-intensity activities at work | *N* | 61 | 61 | 61 | 60 | 60 | 60 | 60 |
|  | ρ | -0.150 | -0.096 | -0.171 | -0.218 | -0.231 | 0.271 | -0.181 |
|  | *P* | 0.248 | 0.461 | 0.187 | 0.095 | 0.076 | **0.037** | 0.167 |
| Time spent walking or bicycling for travel | *N* | 61 | 61 | 61 | 60 | 60 | 60 | 60 |
|  | ρ | -0.237 | -0.234 | -0.339 | -0.385 | -0.318 | 0.336 | -0.250 |
|  | *P* | 0.066 | 0.070 | **0.008** | **0.002** | **0.013** | **0.009** | 0.055 |
| Vigorous-intensity activities at leisure | *N* | 61 | 61 | 61 | 60 | 60 | 60 | 60 |
|  | ρ | -0.059 | -0.006 | -0.034 | -0.157 | -0.142 | 0.178 | -0.102 |
|  | *P* | 0.650 | 0.961 | 0.795 | 0.230 | 0.280 | 0.174 | 0.440 |
| Moderate-intensity activities at leisure | *N* | 61 | 61 | 61 | 60 | 60 | 60 | 60 |
|  | ρ | -0.056 | 0.054 | -0.034 | 0.064 | -0.073 | 0.012 | 0.060 |
|  | *P* | 0.668 | 0.681 | 0.796 | 0.626 | 0.580 | 0.925 | 0.647 |
| Sitting | *N* | 61 | 61 | 61 | 60 | 60 | 60 | 60 |
|  | ρ | 0.027 | 0.072 | 0.077 | -0.022 | 0.095 | -0.082 | -0.047 |
|  | *P* | 0.835 | 0.580 | 0.558 | 0.869 | 0.471 | 0.531 | 0.721 |

*P*<0.05 in bold print. N total number of individuals with data for these parameters.

**Supplemental Table 10.** Spearman correlations between physical activity and body measures in female and male healthy control subjects

|  |  | BMI | Body height | Body weight | Waist circum-ference | Body fat mass | Body muscle mass | Visceral adipose tissue |
| --- | --- | --- | --- | --- | --- | --- | --- | --- |
| Females |  |  |  |  |  |  |  |  |
| Total physical activity | *N* | 31 | 31 | 31 | 28 | 29 | 29 | 29 |
|  | ρ | 0.131 | 0.207 | 0.211 | 0.037 | 0.039 | 0.020 | -0.064 |
|  | *P* | 0.481 | 0.265 | 0.256 | 0.851 | 0.839 | 0.917 | 0.742 |
| Vigorous-intensity activities at work | *N* | 31 | 31 | 31 | 28 | 29 | 29 | 29 |
|  | ρ | 0.130 | 0.202 | 0.176 | 0.001 | 0.164 | -0.121 | 0.182 |
|  | *P* | 0.485 | 0.275 | 0.342 | 0.997 | 0.397 | 0.532 | 0.343 |
| Moderate-intensity activities at work | *N* | 31 | 31 | 31 | 28 | 29 | 29 | 29 |
|  | ρ | -0.097 | 0.119 | -0.017 | -0.051 | -0.068 | 0.122 | -0.009 |
|  | *P* | 0.604 | 0.525 | 0.926 | 0.795 | 0.726 | 0.529 | 0.962 |
| Time spent walking or bicycling for travel | *N* | 31 | 31 | 31 | 28 | 29 | 29 | 29 |
|  | ρ | 0.206 | 0.097 | 0.278 | 0.131 | 0.316 | -0.341 | 0.084 |
|  | *P* | 0.266 | 0.603 | 0.130 | 0.507 | 0.095 | 0.070 | 0.665 |
| Vigorous-intensity activities at leisure | *N* | 31 | 31 | 31 | 28 | 29 | 29 | 29 |
|  | ρ | -0.001 | -0.138 | -0.128 | -0.211 | -0.229 | 0.221 | -0.271 |
|  | *P* | 0.996 | 0.461 | 0.493 | 0.281 | 0.232 | 0.250 | 0.155 |
| Moderate-intensity activities at leisure | *N* | 31 | 31 | 31 | 28 | 29 | 29 | 29 |
|  | ρ | 0.130 | -0.155 | -0.004 | -0.263 | -0.050 | 0.051 | 0.161 |
|  | *P* | 0.484 | 0.404 | 0.982 | 0.176 | 0.798 | 0.793 | 0.403 |
| Sitting | *N* | 31 | 31 | 31 | 28 | 29 | 29 | 29 |
|  | ρ | -0.301 | 0.101 | -0.269 | -0.019 | -0.123 | 0.114 | -0.324 |
|  | *P* | 0.099 | 0.588 | 0.144 | 0.925 | 0.525 | 0.556 | 0.087 |
| Males |  |  |  |  |  |  |  |  |
| Total physical activity | *N* | 30 | 30 | 30 | 29 | 30 | 30 | 30 |
|  | ρ | 0.246 | 0.220 | 0.335 | 0.132 | 0.227 | -0.250 | 0.136 |
|  | *P* | 0.190 | 0.243 | 0.070 | 0.496 | 0.227 | 0.183 | 0.474 |
| Vigorous-intensity activities at work | *N* | 30 | 30 | 30 | 29 | 30 | 30 | 30 |
|  | ρ | 0.159 | 0.146 | 0.230 | 0.094 | 0.092 | -0.006 | 0.099 |
|  | *P* | 0.401 | 0.442 | 0.221 | 0.627 | 0.628 | 0.973 | 0.603 |
| Moderate-intensity activities at work | *N* | 30 | 30 | 30 | 29 | 30 | 30 | 30 |
|  | ρ | 0.147 | 0.067 | 0.177 | 0.090 | 0.262 | -0.236 | 0.151 |
|  | *P* | 0.438 | 0.725 | 0.350 | 0.641 | 0.162 | 0.209 | 0.426 |
| Time spent walking or bicycling for travel | *N* | 30 | 30 | 30 | 29 | 30 | 30 | 30 |
|  | ρ | 0.267 | 0.181 | 0.348 | 0.229 | 0.261 | -0.341 | 0.209 |
|  | *P* | 0.154 | 0.340 | 0.059 | 0.233 | 0.163 | 0.065 | 0.268 |
| Vigorous-intensity activities at leisure | *N* | 30 | 30 | 30 | 29 | 30 | 30 | 30 |
|  | ρ | -0.221 | 0.090 | -0.126 | -0.406 | -0.235 | 0.207 | -0.350 |
|  | *P* | 0.241 | 0.636 | 0.506 | **0.029** | 0.210 | 0.272 | 0.058 |
| Moderate-intensity activities at leisure | *N* | 30 | 30 | 30 | 29 | 30 | 30 | 30 |
|  | ρ | 0.158 | -0.077 | 0.114 | 0.197 | 0.245 | -0.288 | 0.290 |
|  | *P* | 0.406 | 0.685 | 0.548 | 0.306 | 0.193 | 0.122 | 0.120 |
| Sitting | *N* | 30 | 30 | 30 | 29 | 30 | 30 | 30 |
|  | ρ | -0.041 | -0.017 | -0.068 | 0.118 | 0.028 | -0.012 | -0.049 |
|  | *P* | 0.829 | 0.927 | 0.723 | 0.542 | 0.883 | 0.950 | 0.799 |

*P*<0.05 in bold print. N total number of individuals with data for these parameters.
